# The engineered single guide RNA structure as a biomarker for gene-editing reagent exposure

**DOI:** 10.1038/s41598-023-37525-y

**Published:** 2023-07-04

**Authors:** Emmarie C. Ryan, Leslie M. Huggins, Joshua D. Podlevsky

**Affiliations:** 1 Molecular and Microbiology, Sandia National Laboratories, Albuquerque, NM 87185 USA; 2 Computational Biology and Biophysics, Sandia National Laboratories, Albuquerque, NM 87185 USA; 3 Present Address: UNM Health Sciences Center, Albuquerque, NM 87106 USA

**Keywords:** Biotechnology, Molecular biology

## Abstract

CRISPR arrays and CRISPR-associated (Cas) proteins comprise a prevalent adaptive immune system in bacteria and archaea. These systems defend against exogenous parasitic mobile genetic elements. The adaption of single effector CRISPR-Cas systems has massively facilitated gene-editing due to the reprogrammable guide RNA. The guide RNA affords little priming space for conventional PCR-based nucleic acid tests without foreknowledge of the spacer sequence. Further impeding detection of gene-editor exposure, these systems are derived from human microflora and pathogens (*Staphylococcus pyogenes*, *Streptococcus aureus*, etc.) that contaminate human patient samples. The single guide RNA—formed from the CRISPR RNA (crRNA) and transactivating RNA (tracrRNA)—harbors a variable tetraloop sequence between the two RNA segments, complicating PCR assays. Identical single effector Cas proteins are used for gene-editing and naturally by bacteria. Antibodies raised against these Cas proteins are unable to distinguish CRISPR-Cas gene-editors from bacterial contaminant. To overcome the high potential for false positives, we have developed a DNA displacement assay to specifically detect gene-editors. We leveraged the single guide RNA structure as an engineered moiety for gene-editor exposure that does not cross-react with bacterial CRISPRs. Our assay has been validated for five common CRISPR systems and functions in complex sample matrices.

## Introduction

Clustered regularly interspaced short palindromic repeats (CRISPR) and CRISPR associated proteins (Cas proteins) comprise an adaptive immune response found across bacteria and archaea species. CRISPR-Cas systems function to defend against exogenous parasitic mobile genetic elements^1,2^. For Type II CRISPR-Cas systems a single effector Cas protein, Cas9, is employed for nucleolytic cleavage of these foreign genetic materials into short fragments. The cleaved DNA fragments are integrated into the CRISPR array within host bacterial chromosome as new spacer sequences interspersed between the short palindromic repeats^3,4^. Spacer sequences are a genetic memory of past foreign genetic material infections and support a more robust immune response against future encounters^5^. The spacer sequences and short palindromic repeats are transcribed and processed into RNA components that are tightly bound by the Cas9 protein to form a stable ribonucleoprotein (RNP) complex^1^.

The spacer and short palindromic repeat form the CRISPR RNA (crRNA) that guides the Cas9 protein to the genetic target. The Type II CRISPR-Cas RNP additionally contains a transactivating RNA (tracrRNA). The 3ʹ-end of the crRNA and 5ʹ-end of the tracrRNA hybridize and form a stem that is recognized by the Cas9 protein^6^. CRISPR-Cas was quickly recognized as a programmable nuclease for gene-editing, where the guiding spacer sequence in the crRNA would only need to be exchanged for almost any genetic loci that harbored a proximal protospacer adjacent motif (PAM) sequence^2,5,7,8^. Correspondingly, the crRNA/tracrRNA/Cas9 system was simplified by fusing the two RNAs into a single guide RNA (sgRNA) resulting in an sgRNA/Cas9 CRISPR-Cas system^9^. This eliminated the complexity associated with expressing two distinct RNAs that required 5ʹ- and 3ʹ-end processing for proper functionality. Thus, the sgRNA has become synonymous with CRISPR-Cas gene-editing.

CRISPR-Cas gene-editing has supplanted previous systems due to the immensely simplier development cycles^10^. Meganucleases (MNs), zinc finger nucleases (ZFNs), and transcription activator-like effector nucleases (TALENs) all require complex, time-intensive, and difficult engineering for genome targeting and editing. Interest in CRISPR-Cas for therapeutics, bioengineering, biofabrication, and bioenergy has remained intensive with no signs of waning. There are numerous active CRISPR-Cas human patient trails for reverting/curing genetic diseases. Countless human and non-human cell lines, model and non-model organisms have been developed to better understand cancer, pulmonary, and autoimmune diseases^11^. Additionally, the nucleolytic cleavage can be abolished to generate a deactivated Cas9 (dCas9). A dCas9 can be fused with transcriptional repressors (CRISPRi), activators (CRISPRa), epigenetic modifiers, and base-editors^12,13^. A partially deactivated Cas9 nickase (nCas9) enzyme can be deployed to further increase CRISPR-Cas specificity—by requiring recognition of two proximal genetic loci—or for prime editing for reverse transcriptase-directed base-substitutions^14,15^. These tools—along with others on the horizon—have maintained the momentum behind CRISPR-Cas as an essential tool that is used across diverse disciplines for investigating biological phenomena and for genetic engineering a broad range of organisms.

The extensive use of CRISPR-Cas within academic, medical, and industrial facilities has massively increased the risk of gene-editor exposure to working personnel. There is a growing need to develop surveillance methods to reliably and accurately detect CRISPR-Cas9 gene-editing reagents. The abundant use of gene-editors has the potential for the accidental contamination of individuals and the environment. Early detection of contamination is needed to monitor for the potential inadvertent release of or exposure to gene-editors. The primary difficulty with detecting accidental exposure to CRISPR-Cas gene-editor reagents is that the prevalent systems for gene-editing are derived from common human pathogens that can often contaminate human patient samples^16,17^. The first deployed—and still most prominent—CRISPR-Cas system for gene-editing is from *Streptococcus pyogenes* with the second most common system from *Staphylococcus aureus*^2,5,11,18^. *S. pyogenes* and *S. aureus* are highly ubiquitous opportunistic human pathogens with approximately 30% of the human population infected yearly^16,17^. Thus, patient samples must be considered contaminated with bacterial CRISPR-Cas unrelated to potential exposure to CRISPR-Cas for gene-editing reagents.

Conventional molecular detection techniques are ill-suited for specifically discerning gene-editing reagents from bacterial CRISPR-Cas. Lateral-flow assays (LFAs), enzyme-linked immunosorbent assays (ELISAs), and other antibody-based detection assays cannot discern gene-editors from bacterial background^19,20^. The bacterial Cas9 protein is overall unmodified—aside from small variable affinity tags and localization signals—when used for gene-editing, thus antibodies raised against Cas9 cannot distinguish gene-editors from bacterial background contaminants. High throughput activity assays for CRISPR-Cas nucleolytic cleavage^21^ have limited utility for surveilling gene-editor exposure. CRISPR-Cas nucleolytic cleavage activity must be carefully preserved during sample preparation, is sensitive to detergents and inhibitors, as well as time sensitive. If activity can be maintained, these assays rely on a fluorescent-quenched DNA target that corresponds to the spacer sequence in the sgRNA. This is complicated and cumbersome for gene-editing libraries that may require hundreds-to-thousands of DNA targets with sequences that may even overlap with naturally present bacterial CRISPR-Cas systems. Additionally, activity-based assays are ineffective for CRISPRi, CRISPRa, epigenetic modifiers, and base-editors that employ a non-nucleolytic dCas9. Moreover, nickases as pairs for enhanced CRISPR-Cas selectivity or fused to reverse transcriptases for prime editing are expected to be problematic as the partially active Cas9 may not cleave the fluorescent-quenched labeled DNA strand. PCR detection suffers from limited priming spaces in the sgRNA^22,23^. The *S. pyogenes* crRNA region in the sgRNA is only 12-nt outside the spacer and *S. aureus* is 14-nt. Adding the adjoining the spacer sequence region for priming is problematic for libraries with highly diverse spacer sequences. Thus, a novel approach is required to specifically detect gene-editing reagents from bacterial background contamination.

Our approach for the detection of CRISPR-Cas9 gene-editing reagents employ RNA structural elements as a distinguishing moiety from bacterial CRISPR-Cas9. The fundamental basis for our RNA structural approach is that sgRNAs are synthetically engineered molecules and characterized by their unique secondary structure. Bacterial crRNA and tracrRNA molecules are complexed by hydrogen bonding, can be denatured, and separated. In contrast, the sgRNA is a fusion of the crRNA and tracrRNA sequences tethered together by a tetraloop. Denaturing the sgRNA merely unfolds the RNA and will not separate either element. We have leveraged this intrinsic property for developing a capture and probe detection assay to specifically detect gene-editing reagents and not bacterial background contamination. Our capture and probe detection assay employs DNA displacement of the crRNA from the tracrRNA affording a probing surface for fluorescence-based detection. We report the ability to detect five distinct species of CRISPR-Cas and our assay functions in complex mock patient samples.

## Methods

### PCR templates for RNA synthesis

DNA templates for RNA in vitro synthesis were generated from overlapping DNA oligos (IDT) that harbored the T7 promoter sequence (5ʹ-TAATACGACTCACTATAG-3ʹ). Single stranded regions of the overlapping DNA oligos was infilled by PCR. For mutagenesis of the sgRNA tetraloop, mutations were introduced into the overlapping DNA oligos used for infill by PCR. PCR was performed in a 25 µL reaction with 0.5 µM DNA oligos, 1 × Q5 Reaction buffer (25 mM TAPS- HCl at pH 9.3, 50 mM KCl, 2 mM MgCl2, and 1 mM β-mercaptoethanol), 0.2 mM each dNTP, and 0.5 U of Q5 DNA Polymerase (NEB). The reactions were initially denatured at 98 °C for 2 min; cycled 30 times between 98 °C for 25 s, 65 °C for 30 s, and 72 °C for 30 s; with a final extension at 72 °C for 2 min. Full-length templates for RNA synthesis was confirmed by agarose gel electrophoresis.

### RNA in vitro synthesis

RNA synthesis was performed with 200 nmoles of purified DNA templates with MEGAshortscript T7 Transcription (Ambion) following the manufacturer’s instructions. Following the 4 h incubation at 37 °C, the reaction was mixed with formamide loading buffer (80% v/v formamide, 0.04% bromophenol blue, 1 mM EDTA, 5 mM Tris–HCl pH 8.0) and heated at 65 °C for 10 min. The denatured RNA was loaded onto an 8 M urea denaturing 4% polyacrylamide gel. RNA from the excised gel slice was eluted in 50% v/v acid phenol and RNA elution buffer (300 mM NaOAc, 0.5 mM EDTA, 25 mM Tris–HCl pH 8.0) overnight with rotation. Following phenol::chloroform extraction, the RNA was ethanol precipitated and resuspended at 10 µM concentration.

### DNA capture and detection

Into 10 µl phosphate buffered saline (Sigma), 5 pmol of the purified sgRNA or annealed crRNA and tracrRNA (Supplemental Table S1) was combined with 10 pmol 5ʹ-biotinylated DNA capture oligo (Supplemental Table S2), incubated at 65 °C for 3 min, followed by slow cooling to room temperature. The RNA/DNA mixture was added to Streptavidin agarose slurry (Pierce) prewashed with phosphate buffered saline, then incubated at 4 °C with rotation for 30 min. A 5ʹ-Cy5 labeled DNA detection oligo (Supplemental Table S2) was added and incubated at 4 °C with rotation for 1 h. The beads were washed thrice with 50 µl phosphate buffered saline, transferred to a black polystyrene flat bottom microplate, and fluorescence intensity measured on a Synergy H4 Plate Reader (BioTek). For sensitivity measurements, a range of RNA concentrations were used from 5 pmol to 156 fmol that followed a linear regression trendline.

### Detection of sgRNA from cell lysate

HEK293 cells (ATCC) were grown in DMEM medium (Gibco) supplemented with 10% FBS, 1 × Penicillin–Streptomycin mix (Lonza), and 5% CO_2_ at 37 °C to 80–90% confluency. Cells were harvested, homogenized in phosphate buffered saline, incubated at 80 °C for 5 min to denature proteins, and the lysate clarified by centrifugation. Protein concentration was measured by BCA protein assay (Pierce). Cell lysate with a final concentration of 0.75 mg/ml was spiked with 5 pmol of the purified sgRNA or annealed crRNA/tracrRNA duplex. The sgRNA detection assay was performed as described above with lysate used in place of the 10 µl phosphate buffered saline.

### Detection of sgRNA from blood

Defibrinated sheep blood (Remel) was diluted 1:10 with phosphate buffered saline and whole cells pelleted by centrifugation. The supernatant was heat denatured at 80 °C for 5 min and clarified by centrifugation. This blood material was spiked with 5 pmol of the purified sgRNA or annealed crRNA/tracrRNA duplex. The sgRNA detection assay was performed as described above with blood material used in place of the 10 µl phosphate buffered saline.

## Results and discussion

The detection of CRISPR-Cas gene-editing reagent exposure is hindered by the cooption from pathogenic bacterial species. The most prevalent CRISPR-Cas gene-editors are from *S. pyogenes* and *S. aureus*. These species are also some of the most common human bacterial infections, with approximately 30% of the human population infected by one of these bacteria on a yearly basis^16,17^. To prevent false-positive results, an assay to specifically detect CRISPR-Cas gene-editing reagent exposure would need to be selectively non-reactive for bacterial CRISPR-Cas. Several antibody-based assays have been developed to immobilize and detect Cas9 for the presence of CRISPR-Cas gene-editing reagents^19,20^. However, the Cas9 protein primary sequence is overall unmodified between the native bacterial and gene-editing systems. While peptide tags are often appended to the N- and C-terminus of the protein, these can vary considerably. Thus, antibodies raised against Cas9 would be unable to selectively detect CRISPR-Cas gene-editing reagents. Activity assays require foreknowledge of the spacer sequence for the creation of appropriate fluorescently labeled target DNA sequences^24^. Moreover, activity assays require timely and gentle preparations to maintain enzymatic activity necessary for detection. This limits the potential for broadly applicable detection for CRISPR-Cas gene-editors.

### DNA displacement for the detection sgRNA

**Figure 1:**
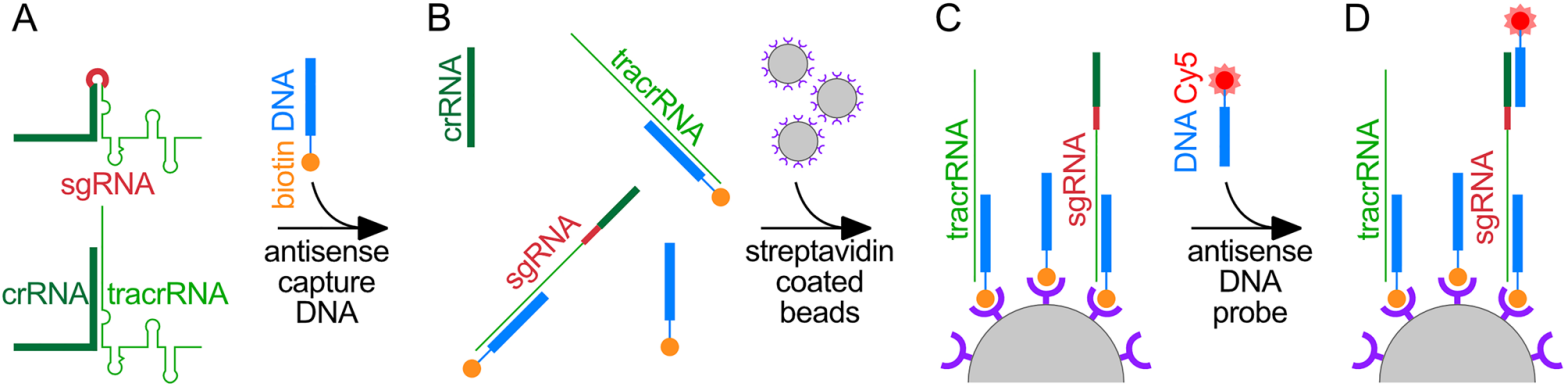
Schematic for the specific detection of the engineered sgRNA indicating gene-editing reagent exposure from the bacterial crRNA and tracrRNA common contaminants. (**A**) The sgRNA is a truncation of the crRNA (dark green) 3ʹ-end and tracrRNA (green) 5ʹ-end fused into a single RNA with a ‘GNRA’ tetraloop (red). (**B**) The capture DNA oligo (blue) harbors a 5ʹ-biotin (orange) and targets the tracrRNA sequence. The tracrRNA-targeting capture DNA unfolds the sgRNA and directly competes with the crRNA/tracrRNA complex, separating the two RNAs. (**C**) The capture DNA-bound sgRNA and tracrRNA are retained by streptavidin beads functioning as a solid support, while the unbound crRNA is washed away. (**D**) The antisense probe DNA oligo harbors a 5ʹ-Cy5 fluorophore (red) and targets the crRNA sequence. The sgRNA is exclusively bound by the probe DNA oligo as the as the crRNA was directly competed away from the tracrRNA.

To overcome these limitations, we investigated the RNA component as a moiety specific for CRISPR-Cas gene-editors and not bacterial contaminants. The RNA component for bacterial CRISPR-Cas comprises two distinct RNAs, the CRISPR RNA (crRNA) and the trans-activating-crRNA (tracrRNA). The most commonly used RNA for CRISPR-Cas9 gene-editing is the engineered single guide RNA (sgRNA)^2,5^. We designed a DNA displacement assay for the specific detection of the sgRNA indicative of gene-editing from the bacterial crRNA and tracrRNA (Fig. 1). For our DNA displacement assay, we designed two distinct DNA oligos. The first DNA oligo functions to capture the sgRNA and maintain an unfolded state. Interaction of the capture DNA with the crRNA and tracrRNA duplex would displace one of the RNAs that would be lost in subsequent wash steps (Fig. 1A,B). The capture DNA is functionalized at the 5ʹ-end with biotin for immobilization onto streptavidin agarose beads that function as a solid support (Fig. 1C). The second DNA oligo is a probe to interrogate the bead-captured RNA. The unfolded sgRNA affords a binding surface for the probe to anneal (Fig. 1D). The crRNA and tracrRNA are antagonistic to the capture and probe DNA oligos and are mutually exclusive for retention on the solid support beads and fluorescence probing for detection.

### Specific detection of gene-editing reagents from bacterial CRISPRs

The *Streptococcus pyogenes* (spy) sgRNA harbors a 2-nt and 4-nt asymmetric internal bulge in the stem that is formed between crRNA and tracrRNA regions (Supplemental Fig. S1A–C). We leveraged this difference in sequence space when designing the capture and probe DNA oligos. The capture and probe DNA oligos have perfect base-pairing with the sgRNA (Supplemental Fig. S1D,E). In contrast, annealing these DNA oligos together would have an intrinsic mismatch (Supplemental Fig. S1F). This mismatch is expected to decrease the stability of the DNA/DNA duplex to promote the DNA/RNA heteroduplex. Additionally, the sgRNA stability is partially compromised by a G:U wobble base-pair at the terminus of the crRNA/tracrRNA (Supplemental Fig. S1A–C). The DNA/RNA heteroduplexes formed between the capture DNA and the tracrRNA as well as the probe DNA and the crRNA replace the G:U wobble with a Watson–Crick base-pairs (Supplemental Fig. S1D,E). Together, the DNA/RNA heteroduplexes have an increased number of perfect base-pairings and replace wobble pairings to increase the stability and to better compete with the RNA-RNA duplex formation.

**Figure 2:**
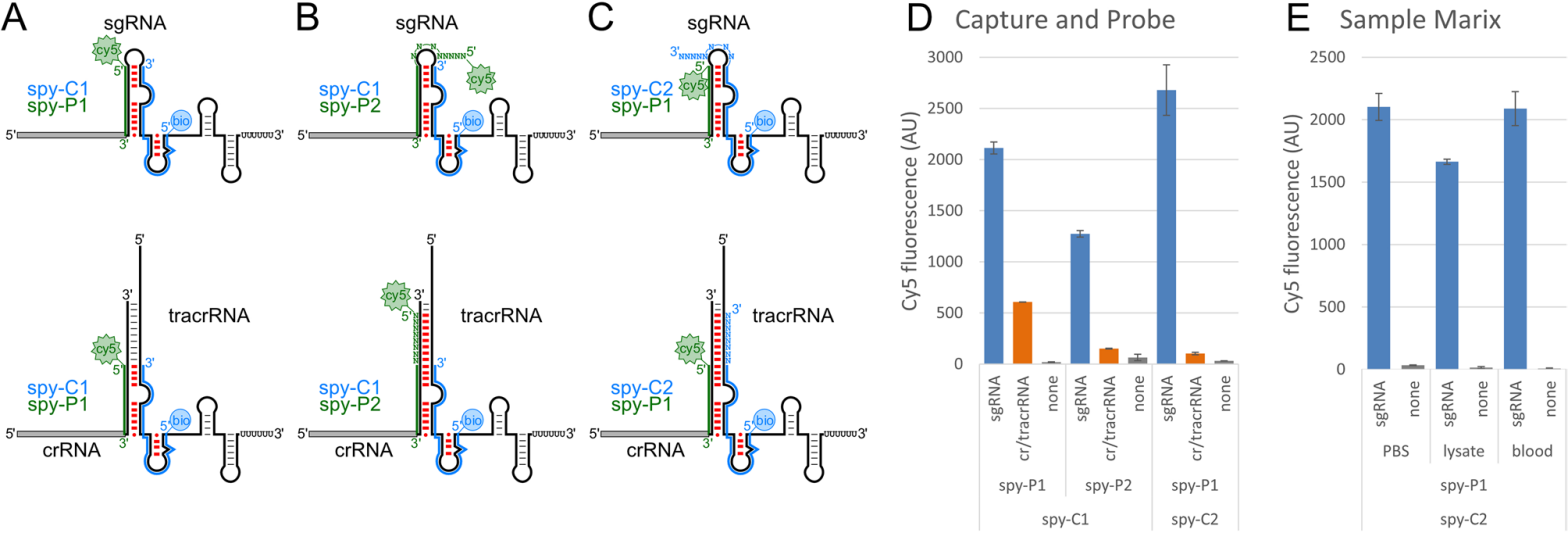
Specific detection of the *S. pyogenes* sgRNA requires a polyN tract to disrupt the crRNA/tracrRNA complex. Schematic of the *S. pyogenes* (spy) sgRNA (top) and crRNA/tracrRNA complex (bottom) with the (**A**) spy-C1 5ʹ-biotin capture DNA oligo (blue) and spy-P1 5ʹ-Cy5 probe DNA oligo (green), (**B**) spy-P2 5ʹ-Cy5 probe DNA oligo (green) with a 5′ 8-mer polyN tract, and (**C**) spy-C2 5ʹ-biotin capture DNA oligo (blue) with a 3′ 8-mer polyN tract. Expected disruption of the base-pairing between the crRNA and tracrRNA regions are denoted (red). (**D**) There was significant Cy5 fluorescence signal from the spy-C1 and -P1 DNA oligos indicating insufficient disassociation of the crRNA from the tracrRNA. Assays with the spy-P2 probe or spy-C2 capture DNA appended with a 5ʹ- or 3ʹ-polyN tract, respectively, had approximately background levels of Cy5 fluorescence for crRNA/tracrRNA complex. (**E**) Detection assay had similar background levels in the presence of HEK293 cell lysate or heat-denatured blood. Error bars are standard error calculated from two independent replicates.

We tested a 5ʹ-biotin labeled capture (spy-C1) DNA oligo that will base-pair with the tracrRNA region of the sgRNA and promote unfolding to expose the crRNA region for detection with the 5ʹ-Cy5 labeled probe (spy-P1) DNA oligo (Fig. 2A). The two heteroduplexes formed between the tracrRNA/capture DNA and crRNA/probe DNA was expected to unpair all interactions between the crRNA and tracrRNA regions in the gene-editor sgRNA. For bacterial CRISPR, the crRNA and tracrRNA have an additional 10-bp of perfect pairings. The capture and probe DNA oligos were not expected to disrupt these additional base-pairs and might allow for the trans RNA-RNA complex to remain intact. To overcome the possibility of the crRNA/tracrRNA trans complex remaining intact, we appended the 3ʹ-end of the capture (spy-C2) DNA oligo (Fig. 2B) and the 5ʹ-end of the probe (spy-P2) DNA oligo (Fig. 2C) with 8-mer polyN tracts. A degenerate polyN tract was chosen in place of a sequence perfect match to allow for sequence modifications to the sgRNA apical tetraloop, aptamer fusions in place of the apical tetraloop, or sequence variations introduced into the 5ʹ-end of the tracrRNA. We analyzed the specificity of detection for the spy-C1/P1, -C1/P2, and -C2/P1 capture and probe pairs (Fig. 2D). The capture DNA was initially added to the sgRNA or crRNA/tracrRNA complex, heat denatured, and then slowly cooled. We omitted the capture and probe pair spy-C2/P2 to avoid non-specific interaction between the two degenerate 8-mer polyN tracts. The spy-C1/P1 oligo DNA pair resulted in a strong Cy5 signal for the sgRNA, yet had a moderate signal for crRNA/tracrRNA. This was likely due to the incomplete disruption of the base-pairings in the trans RNA-RNA complex. In contrast, both the degenerate 8-mer polyN tracts harboring spy-C1/P2, and -C2/P1 oligo DNA pairs decreased the Cy5 signal from crRNA/tracrRNA to near background generated from water alone. Interestingly, the spy-C1/P2 oligo DNA pair generated less signal than the non-degenerate oligo DNA pair yet spy-C2/P1 had a minor increase in signal.

To further explore the sequence space for the capture and probing, we swapped the sequences for capture and probe DNA oligos (Supplemental Fig. S2). Identical sequences were used with the biotin and Cy5 labeled swapped to minimize any changes between experiments (Supplemental Fig. S2A–D). Similarly, we omitted the capture and probe pair spy-C4/P4 to avoid non-specific interaction between the two degenerate 8-mer polyN tracts. The swapped capture and probe DNA oligos maintain the perfect base-pairing with the sgRNA (Supplemental Fig. S3A–C). Annealing the DNA oligos together would have intrinsic mismatches (Supplemental Fig. S3D). We performed the experiment as before, using the biotin DNA oligo for initial capture and probe of the sgRNA or crRNA/tracrRNA complex with the initial heat denaturing and cooling. Unexpectedly, this combination of DNA oligos failed to generate any significant Cy5 signal (Supplemental Fig. S2E). We believe that the lack of signal from the swapped capture and probe DNA oligos was likely due to the capture DNAs. The swapped capture DNA is significantly shorter at 12-nt for spy-C3 compared with 26-nt and 34-nt for spy-C1 and -C2, respectively (Supplemental Figs. S1D and S3B). The shorter spy-C3 capture DNA oligo was likely unable to maintain sufficient interaction with the RNA to maintain an unfolded state for detection with the spy-P3 and -P4 probe DNA oligos.

### Specific detection of gene-editing reagents in complex samples

Detection of gene-editors directly within patient samples affords better avenues for surveillance and reduces samples preparation complexity. However, patient samples are complicated by the presence of lysed cells and blood constituents. We began by evaluating the current sensitivity of our assay within the simple sample media of phosphate buffered saline (Supplemental Fig. S4). The sgRNA was serially titrated 1:2 from 5 pmol down to 156 fmol and fluorescence intensity for the assay measured. Our limit of detection was our lowest value of 156 fmol of sgRNA. To investigate our ability to specifically detect sgRNA from bacterial crRNA/tracrRNA complex in complex sample matrixes, we explored crudely generated human cell lysate and blood. As a mock for collected tissue samples, we used human cells that were crudely lysed in phosphate buffered saline by heating at 80 °C for 5 min and clarified by centrifugation. This method requires little specialized equipment and is amenable to lower resource environments. For the blood sample, the blood was first diluted with phosphate buffered saline and similarly lysed by heating and clarified by centrifugation. In addition to lysing the cells, the heating step would denature proteins and would release RNA from ribonucleoprotein complexes. We spiked these complex sample matrices with the sgRNA to mock exposure to gene-editors. For the detection assay, we employed the spy-C2/P1 oligo DNA pair. While there was a very modest decrease in Cy5 fluorescence for the human cell lysate compared with phosphate buffered saline, the signal was orders of magnitude greater than the control without RNA (Fig. 2E). The small signal decrease was likely due to the steric interference from cellular debris and proteins. These materials likely blocked accessibility of the streptavidin–biotin interaction or the availability of the capture DNA oligo for binding the sgRNA. While the samples had a distinctive coloration from cell lysate and blood, there was no increased background signal. Interestingly, the blood sample did not have any noticeable effect on the Cy5 signal generated by our assay. This demonstrates that out detection assay is amenable to mock patient samples and putatively require minimal sample processing prior to assaying.

### Apical loop and spacer sequence do not impact detection

**Figure 3:**
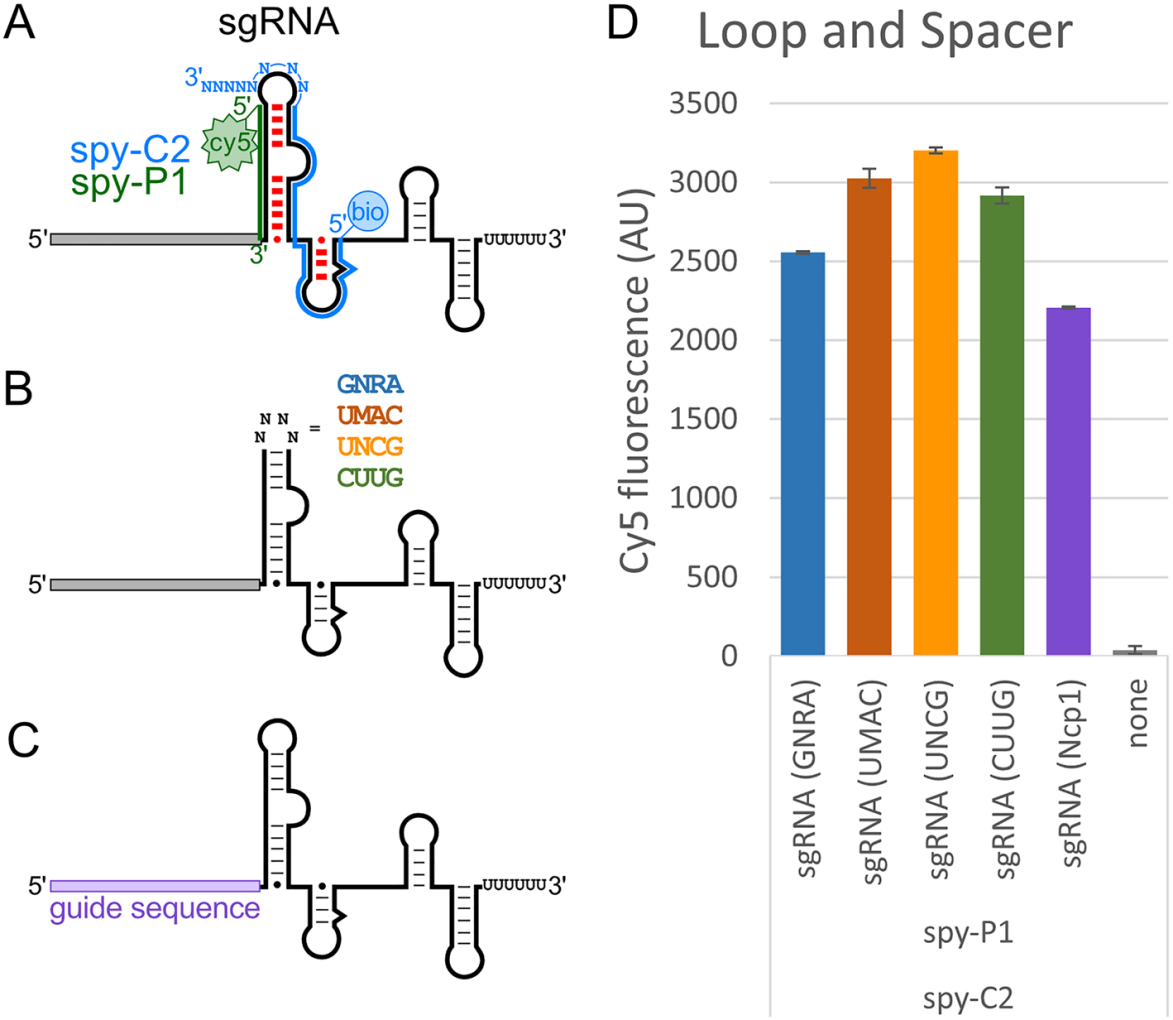
The sgRNA tetraloop and spacer sequences do not affect sgRNA detection. (**A**) Schematic of the *S. pyogenes* sgRNA with the spy-C2 5ʹ-biotin capture DNA oligo 3ʹ-polyN tract (blue) and spy-P1 5ʹ-Cy5 probe DNA oligo (green). Expected disruption of the base-pairing between the crRNA and tracrRNA regions are denoted (red). (**B**) Schematic of the sgRNA ‘GNRA’ (‘GAAA’) tetraloop substituted with ‘UMAC’ (‘UAAC’), ‘UNCG’ (‘UUCG’), and ‘CUUG’ sequences. (**C**) Schematic of the sgRNA with spacer sequence substitutions. In place of a spacer sequence targeting GFP, a spacer targeting the mouse NPC intracellular cholesterol transporter 1 (Npc1), GenBank NM_008720. (**D**) Cy5 intensity as a readout for the detection of the sgRNA with the tetraloop and spacer substitutions. Error bars are standard error calculated from two independent replicates.

Detection of the sgRNA is difficult due to common bacterial contaminants that harbor CRISPR-Cas systems. Further complicating this detection is the method for generating the sgRNA by fusing the crRNA and tracrRNA. The sequence of the crRNA and tracrRNA are truncated within the trans base-paired stem and capped with a tetraloop to fuse the two RNAs into a single RNA (Supplemental Fig. S1A,B). The apical loop sequence for the sgRNA is commonly the ‘GNRA’ tetraloop sequence of ‘GAAA’. However, other tetraloops within the ‘GNRA’ family as well as other degenerate tetraloop sequences that include ‘UMAC’ and ‘UNCG’. Additionally, there have been reports of replacing the tetraloop sequence with aptamer sequences that include MS2, PP7, and boxB^23^. We explored the utility of our 8-mer polyN tract capture DNA for the pull-down of sgRNAs with a variety of different apical loop sequences (Fig. 3). The spy-C2 capture and spy-P1 probe DNA oligos (Fig. 3A) were tested against the default ‘GNRA’ (‘GAAA’) tetraloop sequence along with the ‘UMAC’ (‘UAAC’) and ‘UNCG’ (‘UUCG’) as well as the highly flexible sequence ‘CUUG’ (Fig. 3B). To additionally validate that the spacer sequence did not contribute to detection, we tested another spacer sequence that targeted the mouse NPC intracellular cholesterol transporter 1 (Npc1) gene, GenBank NM_008720 instead of our default target of GFP (Fig. 3C). Interestingly, there was no negative effects from the change in the sgRNA tetraloop sequence (Fig. 3D). Instead, there was a modest increase in fluorescent signal for the other three sequences investigated. The change in the spacer sequence had a very minor decrease in fluorescence signal. Overall, changing the apical loop or spacer sequence did not interfere with our ability to detect the sgRNA.

### Specific detection of additional sgRNAs from other species as gene-editing reagents

CRISPR-Cas from *S. pyogenes* is the most prevalent system for gene-editing, transcription activation and repression. However, gene-editing is limited by the protospacer adjacent motif (PAM) specific sequence that restricts potential cut sites and the size of the Cas9 protein. For *S. pyogenes* CRISPR-Cas, the PAM is ‘NGG’ and Cas9 is 1368-aa protein. Additional CRISPR-Cas systems are being developed as alternatives to *S. pyogenes* that afford more flexibility for the PAM sequence and more critically, employ a smaller Cas9 protein that is easier to deliver to the cell for gene-editing^18^. These other CRISPR-Cas systems are also derived from human pathogens and include other Streptococci species, *S. aureus*, and *Neisseria meningitidis*. *S. aureus* CRISPR-Cas9 is of particular high interest due to the smaller size of the Cas9 protein compared with other systems.

**Figure 4:**
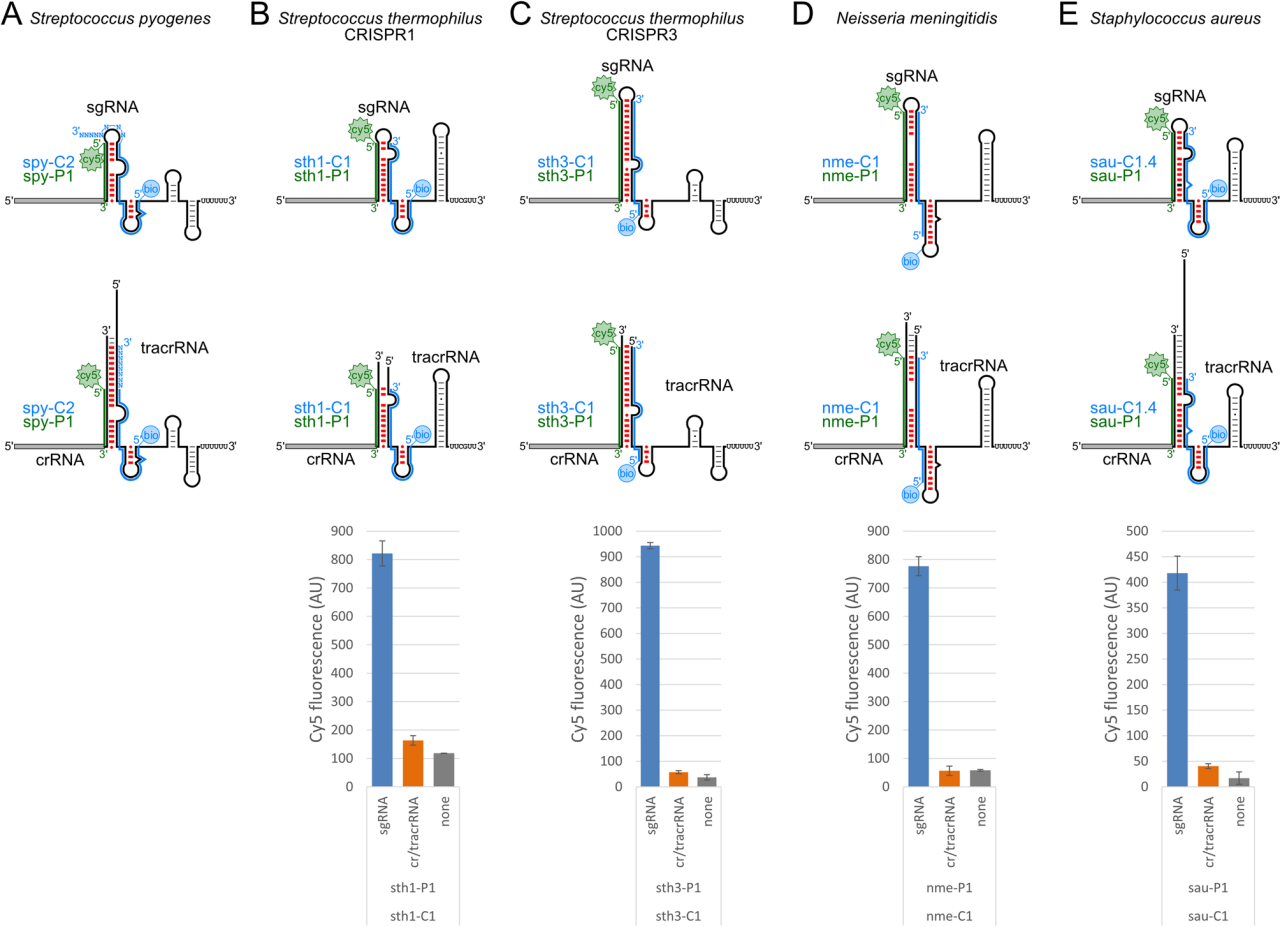
Specific detection of sgRNA from other species. Schematics comparing (**A**) *S. pyogenes* (spy), (**B**) *S. thermophilus* CRISPR1 (sth1), (**C**) *S. thermophilus* CRISPR3 (sth3), (**D**) *N. meningitis* (nme), and (**E**) *S. aureus* (sau) sgRNAs (top) and crRNA/tracrRNA complexes (middle), and assay fluorescence intensity (bottom). The -C1 5ʹ-biotin capture DNA oligo (blue), -P1 5ʹ-Cy5 probe DNA oligo (green), and the expected disruption of the base-pairing between the crRNA and tracrRNA regions are denoted (red). The *S. aureus* capture DNA oligo C-1.4 harbors a single point base-substitution located at the fourth base-pair from the base of the crRNA/tracrRNA stem (black). Error bars are standard error calculated from two independent replicates.

To investigate our ability to detect other species sgRNA from the corresponding crRNA and tracrRNAs, we designed capture and probe DNAs (Fig. 4). We targeted *S. thermophilus* (sth), *N. meningitidis* (nme), and *S. aureus* (sau). *S. thermophilus* is closely related to *S. pyogenes*, yet, has distinct secondary structures and sequence space to explore for our capture and probe assay (Fig. 4A–C). Additionally, *S. thermophilus* has two CRISPR-Cas9 systems termed ‘CRISPR1’ (sth1) and ‘CRISPR3’ (sth3). The *S. thermophilus* CRISPR1 and CRISPR3 systems have distinct crRNA and tracrRNA as well as corresponding sgRNA sequences. Each has been independently developed for gene-editing with unique sgRNAs^25,26^. Unlike *S. pyogenes*, the secondary structures for *S. thermophilus* and *N. meningitidis* crRNA/tracrRNA complexes did not have any extended base-pairing compared with their corresponding sgRNAs (Fig. 4C,D). The *S. aureus* crRNA/tracrRNA complex has an additional eight base-pairs compared with the sgRNA stem (Fig. 4E). However, this is entirely composed A:U base-pairs that are expected to have low structural integrity alone. Thus, we designed the capture DNAs without any polyN tracts as there was no extended crRNA/tracrRNA base-pairs to target. We performed a similar capture and probe assay as for *S. pyogenes* and had low background signal for the crRNA/tracrRNA from *S. thermophilus* CRISPR1, CRISPR3, and *N. meningitidis* (Fig. 4B–D).

In contrast, our initial effort for *S. aureus* sgRNA failed and we had four-fold greater signal from the background control without RNA than from the sgRNA (Supplemental Fig. S5). We postulated that this was due to the greater potential base-parings between the sau-C1 capture and sau-P1 probe DNA oligos (Supplemental Fig. S6). The *S. aureus* crRNA/tracrRNA complex has a longer tract of base-pairs before a smaller internal bulge (Supplemental Fig. S6A–C). Additionally, the terminus of this longer base-paired tract lacks the G:U wobble pair found in *S. pyogenes*. Together, this enhances the association between the sau-C1 capture and sau-P1 probe DNA oligos (Supplemental Fig. S6D–F). We designed variants of the sau-C1 capture DNA oligo that harbored single point base-substitutions (sau-C1.1 to sau-C1.5) for mismatches to reduce hybridization to the sau-P1 probe DNA oligo (Supplemental Fig. S5B–F). The Gibbs free energy for the 9 base-pairings between the sau-C1 capture and sau-P1 probe DNA oligos was calculated using IDT OligoAnalyzer tool. For the perfect match of this tract, the Gibbs free energy was − 12.04. The position of a mismatch within this tract reduced the Gibbs free energy, with the lowest for sau-C1.4 at − 4.86. There was a clear trend found with the position of the single point base-substitutions. The sau-C1.1 capture DNA reduced the background signal levels, while sau-C1.2 had greater affinity for the sgRNA than the probe DNA. However, sau-C1.3, -C1.4, and -C1.5 reduced the background signal the most. We then performed our capture and probe assay with sau-C1.4 to detect *S. aureus* sgRNA from the corresponding crRNA/tracrRNA complex (Fig. 4E) and found a very clear signal for the sgRNA, while crRNA/tracrRNA was approximately background levels.

## Conclusions

The specific detection of CRISPR-Cas for gene-editing is a non-trivial task. Gene-editing reagents have been derived from human pathogens or environmental bacteria that commonly contaminate human patient samples. Conventional molecular detection assays are expected to be non-selective and generate false-positive results in the presence of bacterial contamination. Activity assays require gentle and timely sample preparations, foreknowledge of the spacer sequence for a surrogate DNA, and are unsuitable for deactive dCas9 employed for transcription modulation. We explored employing the RNA secondary structure as a moiety specific for CRISPR-Cas gene-editing reagents. We have developed a capture and probe pull-down and detection assay that specifically detects the sgRNA as a signature for gene-editing reagent exposure. Our assay leverages DNA displacement of the RNA secondary structure in direct competition of the critical trans stem formed between the crRNA and tracrRNA. This competitive DNA displacement unfolds the sgRNA to expose a probing surface, while displacing the crRNA from the tracrRNA that is washed away. We have demonstrated our assay can discriminate sgRNA from crRNA/tracrRNA for five distinct CRSIPR-Cas species, functions within complex samples matrices, and could be employed for surveillance of laboratory personnel that routinely work with gene-editing reagents.

### Statistical information

All assays performed with minimally two independent replicates as stated in the figure legends.

## Supplementary Information

Supplementary Information.

## Data Availability

All data generated or analyzed during this study are included in this published article and the supplementary information files.
