# Supplementary material for: The engineered single guide RNA structure as a biomarker for gene-editing reagent exposure
Source: Sci Rep. 2023 Jul 4;13:10804. doi: 10.1038/s41598-023-37525-y (PMC10319717; doi:10.1038/s41598-023-37525-y)
Supplement: Supplementary file 1 — Supplementary Information. [file 41598_2023_37525_MOESM1_ESM.docx]

**Supplementary figures and table legends**


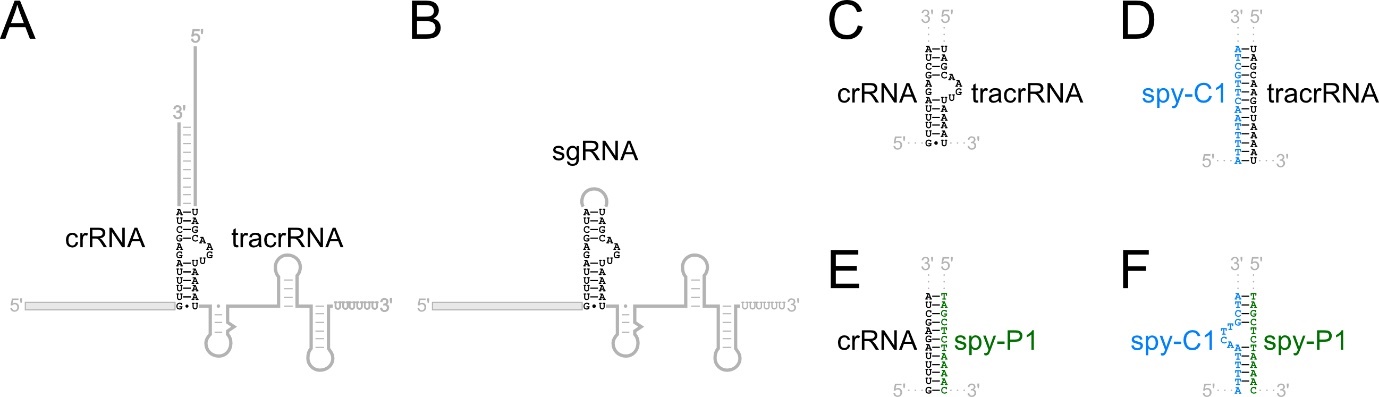


**Supplementary Figure S1.** **Analysis of the *S. pyogenes* asymmetric internal bulge and stem formed between crRNA and tracrRNA.** Schematic of the (A) crRNA/tracrRNA complex and (B) sgRNA with the sequence of the common stem denoted. (C) The internal bulge is asymmetric and composed of 2-nt and 4-nt unpaired. (D) The spy-C1 capture DNA oligo perfectly base-pairs across the 14-nt tracrRNA of the shown region with 26-nt in total, while (E) the spy-P1 probe DNA oligo has only 12-nt of base-pairing. (F) Attempted base-pairing the spy-C1 capture and spy-P1 probe DNA oligos would result in a complementary 2-nt and 4-nt unpaired asymmetric internal bulge that is expected to be highly unstable.


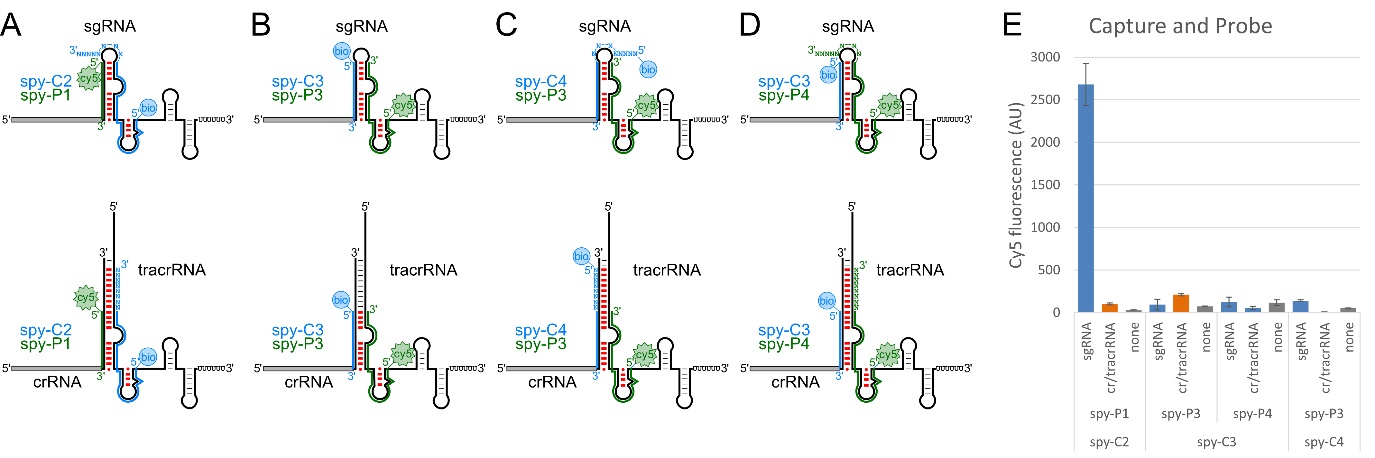


**Supplementary Figure S2. Swapped capture and probe DNA oligos fail to pull down the *S. pyogenes* sgRNA or crRNA/tracrRNA complex.** Schematic of the *S. pyogenes* (spy) sgRNA (top) and crRNA/tracrRNA complex (bottom) with the (A) spy-C2 5’-biotin capture DNA oligo (blue) with a 3’ 8-mer polyN tract and spy-P1 5’-Cy5 probe DNA oligo (green) used in Fig. 2. Swapped capture and probe DNA oligos annealed to the RNA targets with (B) spy-C3 5’-biotin capture DNA oligo (blue) and spy-P3 5’-Cy5 probe DNA oligo (green). The swapped DNA oligos are appended with a (C) 5’ 8-mer polyN tract for the capture DNA oligo (blue) and (D) 3’ 8-mer polyN tract for the probe DNA oligo (green). (D) There was no significant fluorescence signal for any of the swapped capture and probe DNA oligos. Error bars are standard error calculated from two independent replicates.


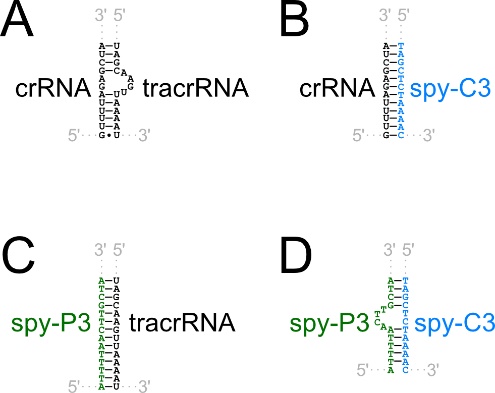


**Supplementary Figure S3. Analysis of the *S. pyogenes* asymmetric internal bulge and stem for the swapped capture and probe DNA oligos.** (A) Schematic of the internal asymmetric bulge composed of 2-nt and 4-nt unpaired. (B) The spy-C3 capture DNA oligo perfectly base-pairs across the 12-nt crRNA region in total, while (C) the spy-P3 probe DNA oligo has 14-nt of base-pairing with the shown tracrRNA region and 26-nt in total. (D) Attempted base-pairing the spy-C3 capture and spy-P3 probe DNA oligos would result in a complementary 2-nt and 4-nt unpaired asymmetric internal bulge that is expected to be highly unstable.


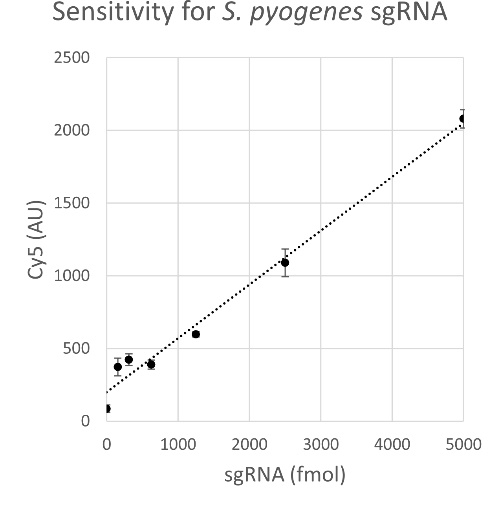


**Supplementary Figure S4. Pull-down assay sensitivity for *S. pyogenes* sgRNA.** The sgRNA was serial titrated at 1:2 dilutions to use 5 pmol down to 156 fmol. The standard capture and probe assay was performed and fluorescence measured. Error bars are standard error calculated from three independent replicates.


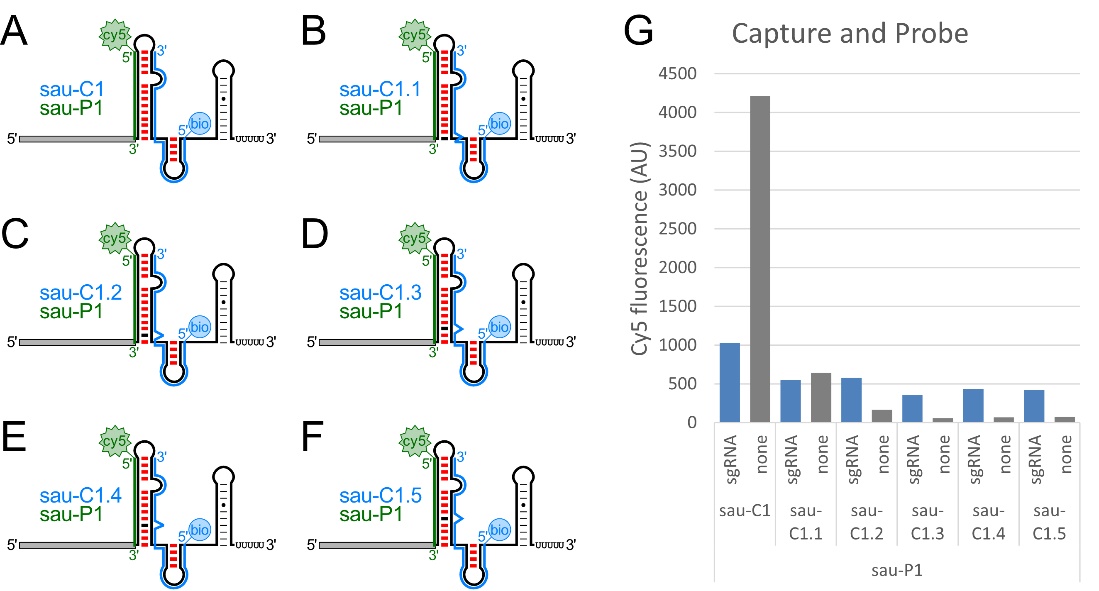


**Supplementary Figure S5. Positional effects of a single point base-substitution mismatch located in the base of the crRNA/tracrRNA stem on capture and probe assay.** The initial (A) sau-C1 capture DNA oligo had a base-substitution to generate a mismatch at (B) the first position for sau-C1.1, (C) the second position for sau-C1.2, (D) the third position for sau-C1.3, (E) the fourth position for sau-C1.4, and (F) the fifth position for sau-C1.5 relative to the terminus of the stem. (G) The standard capture and probe assay was performed and fluorescence measured. The single point base-substitution mismatch was intended to increase the disassociation between the sau-C1 capture and the sau-P1 probe DNA oligos.


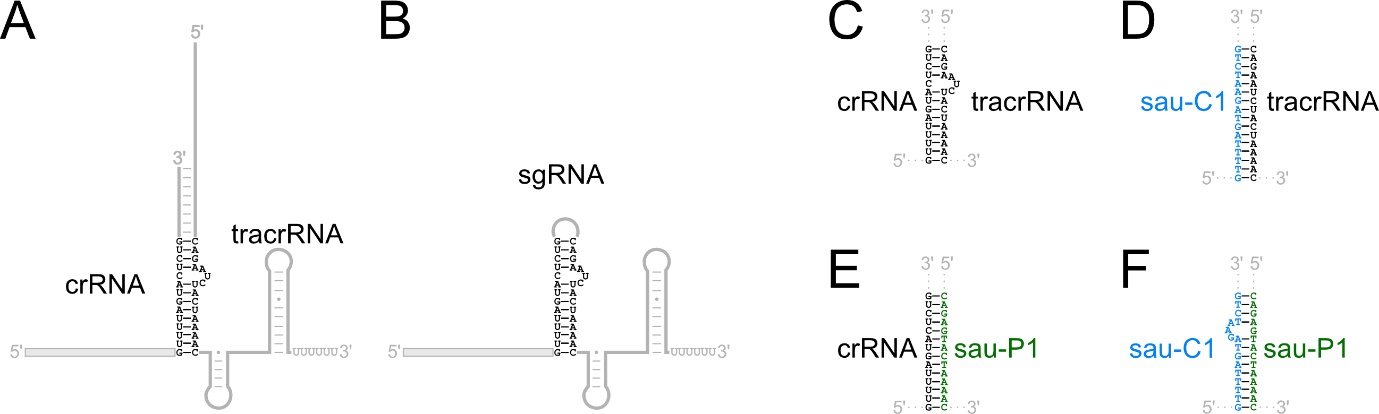


**Supplementary Figure S6. Analysis of the *S. aureus* asymmetric internal bulge and stem formed between crRNA and tracrRNA.** Schematic of the (A) crRNA/tracrRNA complex and (B) sgRNA with the sequence of the common stem denoted. (C) The internal bulge is asymmetric and composed of 1-nt and 3-nt unpaired. (D) The sau-C1 capture DNA oligo perfectly base-pairs across the 16-nt tracrRNA of the shown region with 30-nt in total, while (E) the spy-P1 probe DNA oligo has only 14-nt of base-pairing. (F) Attempted base-pairing the spy-C1 capture and spy-P1 probe DNA oligos would result in a complementary 1-nt and 3-nt unpaired asymmetric internal bulge that is offset to one side of the stem.

**Supplemental Table 1. Sequences for the sgRNA, crRNA, and tracrRNA.** Lowercase ‘g’ indicates non-natural guanosine resides added for *in vitro* transcription.

| **Name** | **Sequence** | **Length** |
| --- | --- | --- |
| spy-sgRNA | **GGGGCCACUAGGGACAGGAUGUUUUAGAGCUAGAAAUAGCAAGUUAAAAUAAGGCUAGUCCGUUAUCAACUUGAAAAAGUGGCACCGAGUCGGUGCUUUUUU** | 102 |
| spy-crRNA | **GGGGCCACUAGGGACAGGAUGUUUUAGAGCUAUGCUGUUUUG** | 42 |
| spy-tracrRNA | **ggGUUGGAACCAUUCAAAACAGCAUAGCAAGUUAAAAUAAGGCUAGUCCGUUAUCAACUUGAAAAAGUGGCACCGAGUCGGUGCUUUUUU** | 90 |
| sau-sgRNA | **GGGGCCACUAGGGACAGGAUGUUUUAGUACUCUGGAAACAGAAUCUACUAAAACAAGGCAAAAUGCCGUGUUUAUCUCGUCAACUUGUUGGCGAGAUUUUU** | 101 |
| sau-crRNA | **GGGGCCACUAGGGACAGGAUGUUUUAGUACUCUGUAAUUUUA** | 42 |
| sau-tracrRNA | **GGGAUUGUACUUAUACCUAAAAUUACAGAAUCUACUAAAACAAGGCAAAAUGCCGUGUUUAUCUCGUCAACUUGUUGGCGAGAUUUUU** | 88 |
| sth1-sgRNA | **GGGGCCACUAGGGACAGGAUGUUUUUGUACUCGAAAGAAGCUACAAAGAUAAGGCUUCAUGCCGAAAUCAACACCCUGUCAUUUUAUGGCAGGGUGUUUUCGUU** | 104 |
| sth1-crRNA | **GGGGCCACUAGGGACAGGAUGUUUUUGUACUCUCAAG** | 37 |
| sth1-tracrRNA | **gggUGCAGAAGCUACAAAGAUAAGGCUUCAUGCCGAAAUCAACACCCUGUCAUUUUAUGGCAGGGUGUUUUCGUU** | 75 |
| sth3-sgRNA | **GGGGCCACUAGGGACAGGAUGUUUUAGAGCUGUGUUGUUUGUUAAAACAACACAGCGAGUUAAAAUAAGGCUUAGUCCGUACUCAACUUGAAAAGGUGGCACCGAUUCGGUGUUUUU** | 117 |
| sth3-crRNA | **GGGGCCACUAGGGACAGGAUGUUUUAGAGCUGUGUUGUUUCG** | 42 |
| sth3-tracrRNA | **GGGCGAAACAACACAGCGAGUUAAAAUAAGGCUUAGUCCGUACUCAACUUGAAAAGGUGGCACCGAUUCGGUGUUUUU** | 78 |
| nme-sgRNA | **GGGAAAUGAGAACCGUUGCUACAAUAAGGCCGUCUGAAAAGAUGUGCCGCAACGCUCUGCCCCUUAAAGCUUCUGCUUUAAGGGGCUUUUU** | 91 |
| nme-crRNA | **GGGGCCACUAGGGACAGGAUGUUGUAGCUCCCUUUCUCAUUUCG** | 44 |
| nme-tracrRNA | **GGGGCCACUAGGGACAGGAUGUUGUAGCUCCCUUUCUCGAAAGAGAACCGUUGCUACAAUAAGGCCGUCUGAAAAGAUGUGCCGCAACGCUCUGCCCCUUAAAGCUUCUGCUUUAAGGGGCUUUUU** | 126 |

**Supplemental Table 2. Sequences of the capture and probe DNA oligos.**

| **Name** | **Sequence** | **5'-modification** |
| --- | --- | --- |
| spy-C1 | **CGGACTAGCCTTATTTTAACTTGCTA** | /5Biosg/ |
| spy-P1 | **TAGCTCTAAAAC** | /5Cy5/ |
| spy-C2 | **CGGACTAGCCTTATTTTAACTTGCTANNNNNNNN** | /5Biosg/ |
| spy-P2 | **NNNNNNNNTAGCTCTAAAAC** | /5Cy5/ |
| spy-C3 | **TAGCTCTAAAAC** | /5Biosg/ |
| spy-P3 | **CGGACTAGCCTTATTTTAACTTGCTA** | /5Cy5/ |
| spy-C4 | **NNNNNNNNTAGCTCTAAAAC** | /5Biosg/ |
| spy-P4 | **CGGACTAGCCTTATTTTAACTTGCTANNNNNNNN** | /5Cy5/ |
| sth1-C1 | **CGGCATGAAGCCTTATCTTTGTAGCTTC** | /5Biosg/ |
| sth1-P1 | **GAGTACAAAAAC** | /5Biosg/ |
| sth3-C1 | **GCCTTATTTTAACTCGCTGTGTTGTTT** | /5Biosg/ |
| sth3-P1 | **CAGCTCTAAAAC** | /5Biosg/ |
| nme-C1 | **CAGACGGCCTTATTGTAGCAACGGTTCTC** | /5Biosg/ |
| nme-P1 | **GGGAGCTACAAC** | /5Biosg/ |
| sau-C1 | **CGGCATTTTGCCTTGTTTTAGTAGATTCTG** | /5Cy5/ |
| sau-C1.1 | **CGGCATTTTGCCTTTTTTTAGTAGATTCTG** | /5Biosg/ |
| sau-C1.2 | **CGGCATTTTGCCTTGCTTTAGTAGATTCTG** | /5Cy5/ |
| sau-C1.3 | **CGGCATTTTGCCTTGTCTTAGTAGATTCTG** | /5Biosg/ |
| sau-C1.4 | **CGGCATTTTGCCTTGTTCTAGTAGATTCTG** | /5Cy5/ |
| sau-C1.5 | **CGGCATTTTGCCTTGTTTCAGTAGATTCTG** | /5Biosg/ |
| sau-P1 | **CAGAGTACTAAAAC** | /5Cy5/ |
